# Evaluating automatic hand hygiene monitoring systems: A scoping review

**DOI:** 10.1016/j.puhip.2022.100290

**Published:** 2022-06-25

**Authors:** Cynthia Tseng, Xueying He, Wenlin Chen, Chung-Li Tseng

**Affiliations:** a Johns Hopkins University Applied Physics Laboratory, Maryland, USA; b University of Electronic Science and Technology of China, Shenzhen, China; c University of Electronic Science and Technology of China, Chengdu, China; d University of New South Wales, Sydney, Australia

**Keywords:** Automatic monitoring system, Hand hygiene, Healthcare-associated infections, Infection control, Scoping review

## Abstract

**Objectives:**

To categorize the extant automatic hand hygiene monitoring systems (AHHMSs) and evaluate the capacity of each to provide information on compliance rates.

**Study design:**

Scoping review.

**Methods:**

Three international databases, PubMed, CINAHL, and EBSCO (between 1 January 2010 and 31 December 2020), were searched according to predetermined inclusion criteria for the scoping review. Two authors screened studies for eligibility independently. The review was conducted according to the Preferred Reporting Items for Systematic Reviews and Meta-Analyses extension for Scoping Reviews (PRISMA-ScR).

**Results:**

Twenty-seven studies were included. Three types of AHHMSs were identified: Type I provides information about the aggregate hand hygiene events (HHEs) only, while Type II adds aggregate hand hygiene opportunities (HHOs), and Type III presents both HHEs and HHOs for individuals. Results suggested that improving the accuracy of recording HHEs and/or HHOs was critical for improving the accuracy of the compliance, which could increase the acceptability of the monitoring system. In addition, the studies found that the implementation of AHHMSs, especially with prompt reminders or additional interventions, could improve the compliance significantly.

**Conclusions:**

The extant AHHMSs could be decomposed into components of 3Ps (product usage monitoring, position monitoring, and performance monitoring). By identifying devices and technology as well as the type of information provided for each component, our approach can aid healthcare facilities to choose a suitable AHHMS that meets their criteria.

## 1 Introduction

Healthcare-associated infections (HAIs) pose a serious public health threat to patient safety. According to a report published by the Agency for Healthcare Research and Quality (AHRQ), HAIs are one of the top 10 leading causes of death in the United States, totaling more than 98,000 deaths each year [1]. The World Health Organization (WHO) reports that 10% of patients in developing countries, compared to 7% of patients in developed countries, acquire at least one nosocomial infection in their lifetime [2].

As a simple procedure, hand hygiene conducted by healthcare workers (HCWs) has been proven theoretically and practically to prevent nosocomial infections [3]. HCWs' overall hand hygiene compliance is generally calculated as the number of hand hygiene events (HHEs) divided by the number of hand hygiene opportunities (HHOs). According to the CDC guidelines, an HHO is defined as one of the “Five Moments for Hand Hygiene” described by the WHO; these include moments before and after touching a patient, before and after a procedure, and after touching a patient's surroundings. Each HHE refers to the performance of hand hygiene, either with alcohol-based hand rub (ABHR) or soap and water. Although the importance of hand hygiene has been identified, the overall compliance is generally low [4,5]. To address the problem of insufficient hand hygiene compliance, the WHO recommends regular hand hygiene monitoring for the purpose of preventing and controlling nosocomial infections [2].

Once considered a gold standard for hand hygiene monitoring, direct observation by trained observers is still widely used to monitoring HCWs' hand hygiene compliance in a variety of healthcare settings [6]. However, there are several drawbacks of using direct observation, such as biased compliance records due to the Hawthorne effect, relatively short windows for observation, and high operational costs [7]. To overcome these problems, automatic hand hygiene monitoring systems (AHHMSs) have been developed and implemented to record HCWs' hand hygiene compliance. The devices and technologies involved in an AHHMS include electronic counters, pressure sensors on ABHR dispensers, doorway entry/exit monitors, infrared beacons, and/or electronic badges [[8], [9], [10]]. Implementing an AHHMS is a significant decision for a hospital. Several issues should be taken into consideration to identify a suitable AHHMS. Since each hospital is unique and is different in their AHHMS requirements, in terms of feasibility, appropriateness, and effectiveness, a scoping review was conducted with an objective to map the system characteristics in existing studies about AHHMSs to categorize the extant AHHMSs and evaluate the capacity of each to provide information on compliance rates. The specific questions discussed in this scoping review were (1) What functional components are included in an AHHMS? (2) What data can the AHHMS provide? (3) How accurate of a compliance rate can be estimated by the system? (4) What is the effectiveness of an AHHMS in improving HCWs’ hand hygiene compliance?

## 2 Methods

### 2.1 Eligibility criteria

The protocol of this scoping review on the extant AHHMSs was drafted in accordance with the Preferred Reporting Items for Systematic Reviews and Meta-Analyses extension for Scoping Reviews (PRISMA-ScR), which was disseminated via email within the review team.

Studies were included in this review if they involved electronic hand hygiene monitoring systems, were conducted in inpatient settings, reported HCWs’ hand hygiene compliance, and were published in the English language with full text. Studies were not selected in this review if they were conducted in other settings, such as outpatient departments, schools and communities, reported hand hygiene compliance involved in patients or visitors, not HCWs, and were published in other types of studies, e.g., concise communication, brief report, and posters.

### 2.2 Information sources and search strategy

We searched PubMed, CINAHL, and EBSCO for all relevant articles published between 1 January 2010 and 31 December 2020 for the purpose of identifying eligible studies in the review. The keywords for the three different databases are provided in Appendix A. Additional eligible studies were identified from manual reference listing of relevant articles.

### 2.3 Data extraction and quality assessment

Under the supervision by two senior authors (W.C and C-L.T), two authors conducted the process of charting, collating, summarizing and reporting results independently by using a standardized data extraction spreadsheet (C.T. and X.H.). The spreadsheet was piloted on eight randomly selected studies and modified accordingly. A finalized standardized template was used for data extraction that included information about study design, duration, the study setting, devices, and technologies in an AHHMS, the type of data collected, additional interventions, and outcomes. Then, the Mixed Methods Appraisal Tool (MMAT) in Appendix B was used to assess the overall methodological quality of the studies based on four MMAT items. All papers were evaluated by two authors (C.T. and X.H) independently and discrepancies were resolved by consensus and consultation with senior authors (W.C and C-L.T).

### 2.4 Data synthesis

We synthesized the data descriptively to provide information about our questions in this scoping review. We first divided the devices and technologies in extant AHHMSs into three distinct components (3Ps) due to their unique functionalities, based on the authors’ discretion. Then, three types of AHHMSs with different combinations of the components were briefly introduced, followed by an evaluation of each type of AHHMS in the discussion section.

## 3 Results

### 3.1 Study selection

**Fig. 1:**
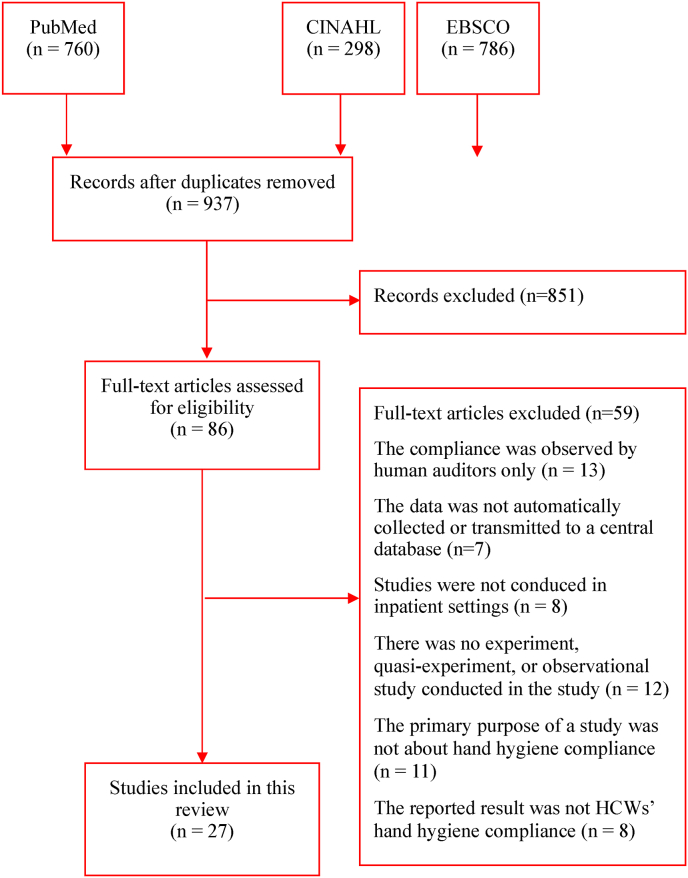
Scoping review process.

Initially 1844 findings were identified from PubMed, CINAHL, and EBSCO. After removing duplicated studies, 937 publications remained for screening. 910 articles were excluded because they did not match the inclusion criteria. Eventually, 27 papers were included in this scoping review. The details of the study selection process are presented in Fig 1.

### 3.2 Study characteristics

By reviewing 27 studies, we divided and categorized the extant AHHMSs into 3Ps that refer to three components: a product usage monitoring component, a position monitoring component, and a performance monitoring component. Seven studies of the AHHMSs included only a product usage monitoring component (the first P), which could only provide information about aggregated HHEs. This system was labeled as a Type I AHHMS. Two studies of the AHHMSs included two components (the first 2Ps), which could provide information about aggregated HHEs and HHOs, and were called Type II AHHMSs. Eighteen studies of the AHHMSs were labeled as Type III AHHMSs, which included components of all 3Ps to automatically detect each individual HCW's HHEs and HHOs.

### 3.3 The 3Ps of an AHHMS

The first P is a product usage monitoring component, usually involving a hand hygiene product dispenser with electronic counting devices or sensors to record whether the product, such as soap solution, chlorhexidine, or alcohol-based hand rub (ABHR), was consumed [11,12]. In addition, the time, location and frequency of HHEs were recorded. A locking mechanism was generally used in the product usage monitoring system to distinguish between multiple presses by the same person for each new HHE [7,13]. For example, multiple presses within a certain short time (e.g., 2s or 3s) were counted and recorded as one HHE.

The second P of an AHHMS, the position monitoring component, was usually installed at the entry or exit of a ward, the ceiling in patient zones, around patient beds, or next to sanitizers, and was used to detect an individual's presence. Three types of technologies, radio-frequency identification (RFID), infrared (IR), and Zigbee, were used. For example, an RFID tag would be worn by an HCW and by communicating through a proprietary wireless sensor protocol, the position of the individual with the tag could be located [14]. To identify thermal infrared energy, two invisible cones were formed to avoid incorrect counting when individuals in a corridor passed by the doorway or individuals in a ward approached the door but did not leave [15]. Zigbee, characterized by its lower power consumption and being less expensive than other technologies, was also used for identifying individuals' positions [16].

A performance monitoring component, the third P of an AHHMS, was used to detect whether HCWs cleaned their hands during each opportunity. Two main approaches were used to validate hand hygiene behavior. The first approach was to monitor whether an HCW washed their hands within a certain period of time, before entering the vicinity of the patient's bed and/or after leaving this area [17]. The second approach was to use chemical sensors installed in wearable devices to detect the use of alcohol gel by the vapor produced [18]. If hand hygiene was not performed within the HHOs, HCWs could receive prompt reminders like a vibration, a visual prompt, or an auditory reminder from their wearable devices.

### 3.4 A type I AHHMS that provides aggregated HHEs

Seven studies used a Type I AHHMS to monitor HCWs' hand hygiene compliance (Table 1). Five of them were prospective observational studies and two were quasi-experimental. The study periods ranged from 384 h [7] to 25 months [19]. As for the study setting, 2 studies monitored HCWs’ compliance in a single unit and 4 studies in multiple units, and one study was in a whole hospital [20]. All studies used a product usage monitoring component to record HHEs through the activation of a hand-rub dispenser.

**Table 1:** Characteristics of studies about a Type I AHHMS.

Author (year)	Study design (duration)	Study setting (location)	Devices and technologies	HHEs identification	HHOs identification	Additional interventions	Major findings about compliance
Azim et al. (2016) [11]	Prospective observational study (13 months)	One 24-bed medical ward and one 20-bed surgical ward in a tertiary teaching hospital (Australia)	ABHR dispensers, electronic counter, low-power radio-relay devices	Activating ABHR dispensers	“Five Moments for Hand Hygiene” by direct observation	No	The overall compliance on the surgical ward was higher compared with the medical ward during weekdays (60% vs 53%, P < 0.05) as well as the weekend (44% vs 34%, P < 0.05); the AHHMS could collect 4 times more the amount of data than human auditors
McLaws et al. (2018) [12]	Prospective observational study (13 months)	One 24-bed medical ward and one 20-bed surgical ward in a tertiary teaching hospital (Australia)	ABHR dispensers, electronic counter, low power radio relay devices	Activating ABHR dispensers	“Five Moments for Hand Hygiene” by direct observation	No	The Hawthorne effect on direct human auditing highly inflated compliance rates; daily hand hygiene ambassadors or reminder technology could harness clinicians' ability to hyperrespond to produce habitual compliance
Hagel et al. (2015) [7]	Prospective observational study (384 h)	One 24-bed ICU at a university hospital (Germany)	ABHR dispensers, electronic counter, Wi-Fi	Pushing the lever of ABHR dispenser	“Five Moments for Hand Hygiene” by direct observation	No	The overall compliance was 51% (95% CI, 49% - 53%); directly and electronically observed HHEs were in agreement
Helder et al. (2012) [21]	Prospective observational study (1 year)	One 27-bed NICU at a university hospital (Netherlands)	ABHR dispensers, electronic counter, wireless transmitting equipment, Wi-Fi	Pushing the lever of an ABHR dispenser	“Five Moments for Hand Hygiene” by direct observation	No	The overall compliance rates were 41%, 34.9%, and 24.1% during day shifts, evening shifts and night shifts, respectively
Diefenbacher et al. (2019) [13]	Quasi-experiment (5 months)	Four non-ICUs in a tertiary hospital (Germany)	ABHR dispensers, electronic counter, Wi-Fi	Activating ABHR dispensers	“Five Moments for Hand Hygiene” by direct observation	Goal setting; collective performance feedback	Single intervention: both feedback and goal setting could improve HHEs slightly during the intervention phase without sustainability; combined interventions: HHEs were nearly doubled when compared to baseline with sustainability
Kwok et al. (2016) [19]	Quasi-experiment (25 months)	One 24-bed medical ward and one 20-bed surgical ward in a tertiary teaching hospital (Australia)	ABHR dispensers, electronic counter, low power 900-MHz radio-relay devices	Activating ABHR dispensers	“Five Moments for Hand Hygiene” by direct observation	Goal setting; nudging	The compliance rates were 85% and 87% on the medical and surgical wards with an overt AHHMS; the compliance dropped to 30% and 49% with a covert AHHMS; on average, compliance during the intervention without being refreshed did not change on the medical ward, whereas the average rate on the surgical ward declined by 9%
Conway et al. (2014) [20]	Prospective observational study (14 months)	A 140-bed community hospital (United States)	ABHR and soap dispensers with electronic counters	Pushing the dispenser	No	Monthly feedback	The compliance ranged from 63.5% to 69.5% before feedback, and from 64% to 78.7% after giving feedback

Azim et al. [11] conducted a prospective observational study in one medical ward and one surgical ward of an 850-bed university teaching hospital in Australia. The ABHR dispensers installed in the corridors and at the bedside were equipped with low power 900-MHz radio relay devices, so that the data about HHEs could be transmitted automatically to the central database once the dispensers were activated. As for the data about HHOs, they were collected by 60 auditors (30 auditors in each ward) over 7 days. There was no additional intervention carried out in this study. The result showed that the overall compliance for HCWs was 48% in the medical ward and 56% in the surgical ward.

Kwok et al. [19] installed the same monitoring system as the one in Azim et al. [11] to study the relationship between supplemental interventions and HCW compliance rates over four phases in a medical ward and a surgical ward in Australia. After the installation of the AHHMS with staff's knowledge, the second phase included aggregate feedback, goal setting, and a community effort to nudge others. Then, the staff were not reminded or given feedback in the third phase. The data showed that during phase 2, compliance rates were similar to or higher than before the intervention and in phase 3, rates were similar to or lower than in phase 2. Researchers concluded that compliance returned to preintervention levels without active intervention, signaling a need for HCW commitment to improvement.

The monitored hand hygiene compliance ranged from 24.1% [21] to 87% [19]. In addition to implementing the AHHMSs, some studies carried out additional interventions, e.g., goal setting and performance feedback. The results showed that additional interventions could help increase compliance to some extent.

### 3.5 A type II AHHMS that provides aggregated HHEs and HHOs

Two studies implemented a Type II AHHMS for the purpose of monitoring HCWs' hand hygiene compliance (Table 2). Boyce et al. [15] conducted a quasi-experimental study to determine the impact of an AHHMS and further interventions on HAI rates. Both direct observation and an AHHMS were used in 4 units; the entrance and exit HHOs and a dispenser's activated HHEs were recorded and shown on a real-time display in each unit. A few more interventions were also carried out, as a goal-setting initiative, bringing in experts that consulted on “frontline ownership,” as well as an intervention involving the support of hospital leadership, and then the adoption of the Toyota Kata performance initiative, which involved changing behavior through leadership and collaboration. The results showed that improvement of compliance was not sustained with the installation of an AHHMS only, but with the implementation of several supplementary interventions, the compliance could be improved by 85%.

**Table 2:** Characteristics of studies about a Type II AHHMS.

Author (year)	Study design (duration)	Study setting (location)	Devices and technologies	HHEs identification	HHOs identification	Additional interventions	Major findings about compliance
Boyce et al. (2019) [15]	Quasi-experiment (3 years)	Four units in a 93-bed nonprofit hospital (United States)	Activity counters, dispenser actuation counters, data receivers, a secure server, a digital monitor	Counting soap dispenser actuation and ABHR dispenser actuation	Monitoring thermal infrared energy when a human body walks through detection zones; direct observation	Goal setting, feedback, hospital leadership support, Toyota Kata methodology	The compliance generated by direct observation was much higher than compliance generated by an AHHMS; the AHHMS only could not guarantee sustained improvement in compliance; the AHHMS with supplementary interventions resulted in a steady and improved compliance (85%); non-Clostridioides difficile HAIs decreased but not in a statistically-significant way
Ellison III et al. (2015) [22]	Quasi-experiment l (25 weeks)	Two medical ICUs at an academic medical center (United States)	Electronic hand hygiene alcohol/soap dispensers, doorway entry/exit monitors	Activating alcohol/soap dispensers	Monitoring room entry/exit events	Real-time feedback, room entry/exit chimes	Unit-wide hand hygiene compliance increased 24% with the use of a combination of room entry/exit chimes and real-time feedback; the performance returned to baseline during the follow-up phase

To investigate the relationship between reminder systems and compliance, Ellison III et al. [22] conducted a controlled clinical trial in two medical ICUs using an AHHMS with reminder systems added. Electronic dispensers for soap and alcohol were paired with entry and exit monitors that were programmed to provide a chime reminder at each HHO. In some intervention phases, an anonymous real-time display of the compliant ratio of HHEs to HHOs was also constantly visible on central computer screens. The studied interventions included combinations of continuous real-time display of compliance on technology, entry and exit chimes, and manager reports. When all three were used, the ratio of HHOs to HHEs increased from 26.1% to 36.6%. However, the performance returned to baseline during the follow-up phase. The researchers concluded that in an ICU, real-time feedback significantly improved hand hygiene activity.

### 3.6 A type III AHHMS that provides individual HHEs and HHOs

Eighteen papers installed a Type III AHHMS (Table 3). Nine publications were prospective observational studies, two were retrospective cohort studies, three were (quasi-)experiments, and four were using mixed methodologies. The study periods lasted from 145 h [23] to 44 months [18]. Five studies were conducted in a single unit, and the other studies were carried out in multiple units. In addition to the components used in the Type II AHHMS to identify HHEs (e.g., monitoring product usage) and HHOs (e.g., recognizing the presence of people), a Type III AHHMS usually included one more component to detect individual hand hygiene performance, such as electronic badges and bracelets. The results showed that wearable devices with prompt reminders, e.g., audio and/or visual reminders, could improve the compliance to as high as 100% [24].

**Table 3:** Characteristics of studies about a Type III AHHMS.

Author (year)	Study design (duration)	Study setting (location)	Devices and technologies	HHEs identification	HHOs identification	Additional interventions	Major findings about compliance
Cheng et al. (2011) [8]	Prospective observational study (3 months)	A 6-bed neurosurgical intensive care unit (Hong Kong Special Administrative Region, China)	Sensors embedded in soap and alcohol dispensers, beacons, electronic badges, proprietary wireless sensor protocol	Activating soap and alcohol dispensers	Sensing proximity to patient zones by beacons; direct observation	No	13,694 HHOs were identified by the system; the overall compliance was 35.1%; the compliance for nurses with shared badges (23.7%) was much lower than the compliance for registered nurses (36.1%) and nursing managers (34.0%) with named badges
Filho et al. (2014) [26]	Prospective observational study (14 weeks)	A 20-bed step-down unit of a tertiary care, private hospital (Brazil)	Electronic counters, ABHR dispensers, bedside sensors, badges with visual reminders, wireless communication protocol	Activating alcohol dispensers	Monitoring HCWs approaching a patient bed; direct observation	No	An AHHMS identified 414 hand hygiene episodes, whereas the human observers identified 448 episodes, resulting in a good accuracy (92%) of the AHHMS
Dyson and Madeo (2017) [27]	Prospective observational study (23 weeks) + interviews	Two units of a large general hospital (United Kingdom)	ABHR dispensers, badges contained in-built alcohol sensors and red, amber and green LEDs, infrared sensors	Cleaning hands with soap and water	Monitoring patient zone entry/exit events	No	Before the installation of the AHHMS, the compliance was 73%; with the installation of the AHHMS, the compliance was 83%; after the system was removed, the compliance went back to 73%; the AHHMS increased HCWs' awareness, enhancing their empathy for patients
McCalla et al. (2017) [9]	Retrospective cohort design (2 years)	One 8-bed ICU and one 25-bed ICU in a community hospital (United States)	ABHR dispensers, electronic badges with visual and audible reminders as well as a chemical sensor, room entry/exit sensors, base station, cloud computing-based applications	Identifying the presence of alcohol	Monitoring room entry/exit; direct observation	No	The compliance detected by the AHHMS was lower than that from human monitors, but the AHHMS did ensure that the hospital met its 95% hand hygiene compliance goal; overall, HAIs dropped substantially during the period that the AHHMS was in use
Iversen et al. (2020) [28]	Prospective observational study (11 months)	One 29-bed unit in a university hospital A and one 17-bed unit in a university hospital B (Denmark)	Sensors located on all ABHR dispensers, name tags of the HCWs, bed sensors to create patient clean zones	Activating ABHR dispensers	Monitoring patient zone entry/exit events; direct observation	No	The overall compliance for nurses from hospital A was 52% and that from hospital B was 36%; nurses sanitized after patient contact more often than before; sanitizers located closest to room exits and in hallways were used most frequently; there was a positive association between the number of sanitations and the compliance levels
Monsalve et al. (2014) [29]	Prospective observational study (10 days)	One 20-bed medical ICU at a large university hospital (United States)	Beacons for position monitoring, beacons for hand hygiene usage, badges, Wi-Fi	Activating ABHR dispensers	Monitoring room enter/exit events	No	Without the presence of other HCWs, the compliance of an HCW was 20.85%; with the presence of other HCWs, the compliance increased significantly to 27.9%; the compliance increased with the number of nearby HCWs but at a decreasing rate
Srigley et al. (2014) [30]	Retrospective cohort study (8 months)	Two multi-organ transplant units at an academic acute care hospital (Canada)	Battery-operated tags, wireless receivers, ABHR and soap dispensers, ultrasound signals	Activating ABHR and soap dispensers	Monitoring patient zone entry/exit events; direct observation	No	HHEs in dispensers visible to auditors were significantly higher than in dispensers not visible to the auditors; the rate increased significantly when auditors were present compared with 1–5 min prior to the auditors' arrival
Marra et al. (2014) [16]	Quasi-experiment (11 months)	Two 20-bed step-down units at a private tertiary care hospital (Brazil)	Electronic handwash counters for alcohol gel, wireless identification badge with visual reminders, wall-mounted sensor, Zigbee system	Pressing the alcohol gel dispensers	Monitoring patient zone enter/exit events	No	A significant increase in the compliance with the installation of the AHHMS (from 74.5 to 90.1 episodes/patient-day); the consumption of ABHR increased too (68.9–103.1 ml/patient-day)
Levchenko (2011) [23]	Prospective observational study (145 h)	Two four-bed and two two-bed rooms on a nursing unit of a complex continuous care hospital (Canada)	Portable and stationary dispensers, badge-sized devices with a microcontroller, infrared detector and short-range radio frequency transceiver, sensors installed at monitored zones	Activating portable and stationary dispensers	Monitoring patient zone enter/exit events	No	With the implementation of the AHHMS, HHEs improved about 53%
Boyce et al. (2019) [14]	Prospective observational study (41 weeks)	One 13-bed surgical ICU (SICU) and one 21-bed general medical ward (GMW) at a university hospital (United States)	Badges, wall-mounted touch-free ABHR dispensers, wireless infrared sensors placed in rooms and hallways, line-of-sight infrared communication protocol,	Activating ABHR dispensers	Monitoring patient room enter/exit events; direct observation	Performance feedback	The average accuracy of the AHHMS was 60%; initiation of the AHHMS was associated with a transient drop in entry and exit compliance on both units
Benudis et al. (2019) [17]	Quasi-experiment (8 months) + survey	Two units at a tertiary care hospital (United States)	Bracelets with vibration and visual reminders, sensors in the soap and waterless hand sanitizer dispensers and above patient beds	Activating sanitizer dispensers	Entering/leaving the vicinity of a patient's bed; direct observation	Performance feedback	HCWs had negative attitudes about the implementation of the AHHMS; the number of hand hygiene events was small due to infrequent bracelet use
Levchenko et al. (2013) [31]	Prospective observational study (2112 h)	One complex continuing care unit of a rehabilitation hospital (Canada)	Beacons attached to the ceiling, wearable badge-like monitors, personal portable and wall-mounted alcohol gel or soap dispensers, infrared and radiofrequency	Activating sanitizer dispensers	Monitoring patient room enter/exit events	Performance feedback	After the automated reminder signal was activated and the nurses started receiving feedback reports, the compliance significantly improved
AI Salman et al. (2015) [24]	Prospective observational study (28 days) + interviews	Two 16-bed coronary care units in a medical facility (Kingdom of Bahrain)	Sensing beacon, dispenser monitors, badges with reminders (red light and vibration), a base and a battery charger, Wi-Fi	Pressing on a bottle of ABHR or soap dispensers	Monitoring room entry/exit events	Leaderboard with most compliant HCWs	The compliance increased to 75% at the end of the 28-day trial; in some cases, the compliance increased to 85% or even 100%
McCalla et al. (2018) [18]	Quasi-experiment (44 months)	All units at a 292-bed community hospital (United States)	ABHR dispensers, electronic badges with visual and audible reminders as well as a chemical sensor, room entry/exit sensors, base station, cloud computing-based applications	Identifying the presence of alcohol	Monitoring room entry/exit	Educational training	On average, the compliance was 95.3% during the intervention period; the use of reminders on the badges was associated with an increased number of hand hygiene events upon room entry; a significant reduction in HAIs was observed
Storey et al. (2014) [25]	Quasi-experiment (6 weeks) + survey	A 96-bed specialist acute cardiac hospital (United Kingdom)	ABHR dispensers, device tagged to beds, overbed tables and chairs, device placed in the waste pipe from ward sinks, identify badge with electronic and visual monitoring, Wi-Fi	Activating ABHR and soap dispensers	Monitoring patient zone enter/exit events	Performance feedback	The compliance increased from 21% to 66% during active immediate feedback; compliance decreased when feedback was provided to wards retrospectively
Gould et al. (2020) [32]	Prospective observational study (15 months)	An acute 31-bed medical ward (United Kingdom)	Antennae mounted in the ward ceiling, tags, Internet- connected ABHR dispensers, cloud-based information technology infrastructure	Activating ABHR	Presenting in a patient zone for at least 10 s	Performance feedback	The 84% agreement between the AHHMS and the manual observation suggest a high level of precision for the evaluated system
Edmisten et al. (2017) [33]	Prospective observational study (17 months)	All inpatient units in three urban/suburban community hospitals (United States)	Wireless radiofrequency communication- enabled badges, position monitoring beacons, hand sanitizer dispensers with monitoring beacons	Activating dispensers	“Five Moments for Hand Hygiene”	Performance feedback	An average monthly hand hygiene compliance was greater than 85%
Fisher et al. (2013) [34]	Experiment (24 weeks)	A cardiology ward and orthopedic and surgical intensive care unit wards from two hospitals (Singapore)	Wireless tag, protection zone transmitters, wash transmitters, wireless reader units	Activating dispensers	Monitoring patient zone enter/exit events; direct observation	Performance feedback	Compliance increased with prompt reminders but was attenuated in the phase with weekly feedback

Storey et al. [25] installed an alcohol-sensing monitoring system across 3 wards. The AHHMS used Wi-Fi and a “traffic light” system for immediate visual feedback. Intervention phases alternated between periods of monitoring without feedback (phase 1), monitoring with feedback, i.e., badges that changed color from green to amber to red if an HHE did not occur (phase 2), no feedback given (phase 3), and feedback given retrospectively using tables and graphs (phase 4) with washout periods between each. When immediate feedback was given, the AHHMS-monitored compliance rose threefold to 66.5% and was maintained into phase 3. During the phase with retrospective feedback, compliance decreased. In this study, automatic monitoring with immediate visual feedback prevented decreases in compliance while feedback given retrospectively did not.

Filho et al. [26] conducted a prospective observational study in a 20-bed unit of a tertiary care hospital in Brazil to examine the accuracy of a Type III AHHMS by comparing the compliance measured by the monitoring system with that measured by direct observation. When measuring the compliance by the Type III AHHMS, HHEs were identified by identification devices (badges) worn by HCWs when they activated electronic alcohol dispensers to clean their hands within 1 min after entering a patient zone. HHOs were identified by the bedside sensors when HCWs approached patient beds. When HCWs approached a patient bed, the badge could trigger the light above the bed. A red light would flash if the HCW did not take hand hygiene measures, while a green light would flash if hand hygiene was performed. Direct observation was conducted by three auditors over a 14-week period. The study showed that the AHHMS found 414 hand hygiene events, while the human observers found 448, indicating a good accuracy of this method (92% overall agreement) (414/448) to monitor HCWs’ hand hygiene behavior.

### 3.7 Efficacy and sustainability

As for the efficacy of the AHHMSs in improving HCWs’ hand hygiene compliance, the reviewed papers showed that there were two approaches to ensure that compliance increased significantly. The first approach was to implement an AHHMS with prompt reminders, which was named as the Type III AHHMS in this paper. The studies showed that prompt reminders, e.g., a blinking red light, an emitting audible tone, and vibrating by electronic badges or bracelets once an HHO was detected, could improve the compliance as high as 100% [24]. The second approach to improve compliance significantly was the implementation of an AHHMS combined with other interventions, e.g., goal setting, leadership support, weekly performance feedback, and educational training [15,17,18].

### 3.8 System acceptance

Several studies considered the acceptance of an AHHMS by HCWs. It was reported that the lack of acceptance of an AHHMS may be because the system failed to accurately record room entries and exits, i.e., the number of HHOs, which would contribute to inaccurate compliance [15]. In addition, the badges could vibrate and/or signal at inappropriate times when there was no HHO, resulting in HCWs’ resistance to embrace the AHHMS [24]. The other concern for HCWs to accept the AHHMS, especially for the Type III AHHMS, was about privacy. Although HCWs welcomed the opportunity to see their own compliance data in order to consider their performance, they worried that the data would be disclosed, resulting in criticism or other undesirable outcomes [27]. In at least two studies involving wearables, HCWs refused to wear devices due to concerns over how collected data would be used and to whom the data would be visible [15,24].

### 3.9 Cost factor

The cost of an AHHMS was reported in one paper reviewed [16]. This paper estimated that the entire cost for a Type III AHHMS was about $50,000 in a 20-bed step-down unit, including installing electronic handwash counters and wall-mounted sensors, preparing badges with visual reminders, and developing the software and system in the unit. Although the other papers reviewed did not report the estimated costs for Type I and Type II AHHMSs, this information could be found in other related papers. One study reported that the cost for installing and maintaining product usage monitoring component (without automatically transferring the data to a central database) in a Type I AHHMS in a 15-bed ICU varied from $30,000 to $40,000 (US dollars), depending on which company was used [35]. Another study reported that 37 Type II AHHMSs installed in outpatient clinics incurred total material costs for full implementation at $12,613 ^36^. Direct observation was often used to obtain the information about HHOs, in order to calculate the corresponding hand hygiene compliance. A 1289-h direct observation study was conducted in outpatient settings, costing $59,210 US dollars [36].

### 3.10 Effect on HAIs

Electronic hand hygiene monitoring has the potential to significantly control and decrease the risk of HAIs. Boyce et al. [15] reports that when supplemental interventions were used, two of four units experienced significant decreases in HAIs. The other two experienced increases in HAIs. Researchers found that the HCWs in the latter two units were inexperienced in using the AHHMS and that the system had a low accuracy. The few studies that report the effect of supplemental interventions on HAI rates were highly biased [32].

## 4 Discussion

Twenty-seven studies using AHHMSs to monitor HCWs' hand hygiene performance were reviewed in this paper. When compared with the extant reviews about AHHMSs [[37], [38], [39]], the strength of our review was the decomposition of AHHMSs into the components of the 3Ps. In understanding the function of each component, the accuracy of measuring HCWs’ hand hygiene compliance could be predicted, and the way to improve the accuracy of the measurement was identified as well. Our approach can aid healthcare facilities to choose a suitable AHHMS that meets their criteria.

Type I AHHMSs automatically captured aggregated HHEs. They usually consisted of a product usage monitoring component that could identify aggregate HHEs. To monitor product usage, automated dispenser count devices [21] were used to record the number of events. Type II AHHMSs could automatically provide information about aggregated HHEs and HHOs. In addition to the component used in the Type I AHHMSs to record HHEs, a position monitoring component was integrated into the Type II AHHMS to capture HHOs, defined as enter/exit patient room/zone [15]. Type III AHHMSs included all three components, i.e., a product usage monitoring component, a position monitoring component, and a performance monitoring component, and could identify each individual's HHEs and HHOs rather than the aggregate.

In addition to using an AHHMS, many studies also conducted direct observation to obtain information about hand hygiene performance, but with different purposes. The main purpose for direct observation in studies using a Type I AHHMS was to obtain information about HHOs, and to calculate overall hand hygiene compliance [11,12]. The calculated compliance ranged from 24.1% to 87% in the studies using a Type I AHHMS. The primary purpose for direct observation in studies using Type II and Type III AHHMSs was to examine the accuracy of the monitoring systems by comparing the data obtained from the AHHMS and that from direct observation. The reported accuracy for the AHHMS to capture HHOs was about 85% [8]. To improve the accuracy of the AHHMS to detect HHOs, calibration should be carried out carefully at a technical development phase to avoid an inaccurate record about HHOs, e.g., multiple entries and exits were recorded when HCWs, in fact, did not enter or exit patient zones. In addition, the locations of position monitoring sensors, e.g., around a patient bed, on the ceiling or above the doorway in a ward, should be carefully considered to avoid missing all the entries and exits. The reported accuracy for the AHHMS to capture HHEs was between 60% and 92% [15,26]. The difference in the accuracy of HHEs occurred during the time period that a sensor recorded an HHE, which ranged from a 2-s period to a 2-min period. If the time period was too long, quickly repeated HHEs were very likely to be misread.

### 4.1 Implications for hospitals

There are some implications for healthcare facilities in choosing AHHMSs to monitor HCWs’ hand hygiene compliance. Each type of AHHMS has advantages and disadvantages in terms of efficacy, accuracy, cost, and acceptance. Before deciding to implement an AHHMS, a healthcare facility should carefully consider their pros and cons. For instance, Type III AHHMSs can improve the hand hygiene compliance in a significant way by requiring HCWs to wear badges or bracelets with prompt reminders. However, HCWs may have concerns about privacy, which can result in a low acceptance of the AHHMS. As a result, greater effort may be necessary in informing HCWs on how data is used, how it is collected, and who has access, for instance, to better balance device acceptance and efficacy. After adequate compliance is achieved, efforts should be taken to maintain heightened compliance rates. Unfortunately, literature has not shown a consistent method to sustain results, thus requiring further study on this topic.

### 4.2 Limitations

While our paper provided a comprehensive review to evaluate existing AHHMSs, there were several limitations to this study. Firstly, although a thorough search was conducted for the literature, it is possible that not all relevant papers were included. Secondly, the papers included in this review were only published in English, which could result in a publication bias in this review. Furthermore, there might be some reporting bias due to the unreported studies with null or negative results.

## 5 Conclusions

To conclude, this paper conducted a comprehensive review about the extant AHHMSs. We decomposed the AHHMSs into components of 3Ps (product usage monitoring, position monitoring, and performance monitoring) such that different combinations of these 3Ps could provide different levels of accuracy for HHEs and HHOs, which are necessary for estimating the compliance rate. By identifying devices and technology as well as the type of information provided for each component, our approach can aid healthcare facilities to choose a suitable AHHMS that meets their criteria. The findings suggest that there is still room to improve the accuracy of AHHMSs. With the improvement of accuracy, HCWs would become more willing to accept AHHMSs. In addition, many studies show that the implementation of AHHMSs, especially with prompt reminders or additional interventions, could improve hand hygiene compliance significantly.

## Declaration of interests

The authors declare that they have no known competing financial interests or personal relationships that could have appeared to influence the work reported in this paper.

## Funding

This work was supported in part by a grant from the 10.13039/501100001809National Natural Science Foundation of China (grant number: 71902017).

## Competing interest

None declared.

## Ethical approval

Not required.
