# Supplementary material for: Evaluating automatic hand hygiene monitoring systems: A scoping review
Source: Public Health Pract (Oxf). 2022 Jun 25;4:100290. doi: 10.1016/j.puhip.2022.100290 (PMC9801014; doi:10.1016/j.puhip.2022.100290)
Supplement: Multimedia component 1 [file mmc1.docx]

Appendix A

| **Database** | **Summarized key words** |
| --- | --- |
| PubMed | Hand [Title/Abstract]) AND (disinfection [Title/Abstract] OR hygiene [Title/Abstract] OR wash [Title/Abstract]) AND (monitor [Title/Abstract] OR technologies [Title/Abstract] OR technology [Title/Abstract] OR automated [Title/Abstract] OR automatic [Title/Abstract] OR surveillance [Title/Abstract]) |
| CINAHL | TI (hand hygiene or handwashing or hand washing or hand disinfection) AND TI (technologies or technology or automated or automatic or surveillance) OR AB (hand hygiene or handwashing or hand washing or hand disinfection) AND AB (technologies or technology or automated or automatic or surveillance) |
| EBSCO | TI (hand hygiene or handwashing or hand washing or hand disinfection) AND TI (technologies or technology or automated or automatic or surveillance) OR AB (hand hygiene or handwashing or hand washing or hand disinfection) AND AB (technologies or technology or automated or automatic or surveillance) |
