# Supplementary material for: Evaluating automatic hand hygiene monitoring systems: A scoping review
Source: Public Health Pract (Oxf). 2022 Jun 25;4:100290. doi: 10.1016/j.puhip.2022.100290 (PMC9801014; doi:10.1016/j.puhip.2022.100290)
Supplement: Multimedia component 2 [file mmc2.docx]

**Appendix B**

Quality assessment by using MMAT

In summary, 16 studies were of high quality (MMAT score > 80%) and 11 studies were of good quality (60% ≤ MMAT score ≤ 80%). The reviewed studies were divided into three categories, i.e., randomized control study, non-randomized study, and quantitative descriptive study. Their average scores were 100% (n = 1), 85% (n = 8), and 94% (n = 18), respectively.

**Table A1. MMAT for randomized controlled study.**

| **Reference** | **MMAT item** | **Yes/No** | **MMAT score** | |
| --- | --- | --- | --- | --- |
| Fisher et al. 2013 | 2.1 Is randomization appropriately performed? | Yes | 100% |  |
|  | 2.2 Are the groups comparable at baseline? | Yes |  |  |
|  | 2.3 Are there complete outcome data? | Yes |  |  |
|  | 2.4 Are outcome assessors blinded to the intervention provided? | Yes |  |  |
|  | 2.5 Did the participants adhere to the assigned intervention? | Yes |  |  |

**Table A2. MMAT for non-randomized study.**

| **Reference** | **MMAT item** | **Yes/No** | **MMAT score** |
| --- | --- | --- | --- |
| Diefenbacher et al. 2019 | 3.1. Are the participants representative of the target population? | Yes | 80% |
|  | 3.2. Are measurements appropriate regarding both the outcome and intervention (or exposure)? | Yes |  |
|  | 3.3. Are there complete outcome data? | Yes |  |
|  | 3.4. Are the confounders accounted for in the design and analysis? | No |  |
|  | 3.5. During the study period, is the intervention administered (or exposure occurred) as intended? | Yes |  |
| Kwok et al. 2016 | 3.1. Are the participants representative of the target population? | Yes | 100% |
|  | 3.2. Are measurements appropriate regarding both the outcome and intervention (or exposure)? | Yes |  |
|  | 3.3. Are there complete outcome data? | Yes |  |
|  | 3.4. Are the confounders accounted for in the design and analysis? | Yes |  |
|  | 3.5. During the study period, is the intervention administered (or exposure occurred) as intended? | Yes |  |
| Boyce et al. 2019 (26) | 3.1. Are the participants representative of the target population? | Yes | 80% |
|  | 3.2. Are measurements appropriate regarding both the outcome and intervention (or exposure)? | Yes |  |
|  | 3.3. Are there complete outcome data? | Yes |  |
|  | 3.4. Are the confounders accounted for in the design and analysis? | No |  |
|  | 3.5. During the study period, is the intervention administered (or exposure occurred) as intended? | Yes |  |
| Ellison III et al. 2015 | 3.1. Are the participants representative of the target population? | Yes | 80% |
|  | 3.2. Are measurements appropriate regarding both the outcome and intervention (or exposure)? | Yes |  |
|  | 3.3. Are there complete outcome data? | Yes |  |
|  | 3.4. Are the confounders accounted for in the design and analysis? | No |  |
|  | 3.5. During the study period, is the intervention administered (or exposure occurred) as intended? | Yes |  |
| Storey et al. 2014 | 3.1. Are the participants representative of the target population? | Yes | 80% |
|  | 3.2. Are measurements appropriate regarding both the outcome and intervention (or exposure)? | Yes |  |
|  | 3.3. Are there complete outcome data? | Yes |  |
|  | 3.4. Are the confounders accounted for in the design and analysis? | Can’t tell |  |
|  | 3.5. During the study period, is the intervention administered (or exposure occurred) as intended? | Yes |  |
| Marra et al. 2014 | 3.1. Are the participants representative of the target population? | Yes | 80% |
|  | 3.2. Are measurements appropriate regarding both the outcome and intervention (or exposure)? | Yes |  |
|  | 3.3. Are there complete outcome data? | Yes |  |
|  | 3.4. Are the confounders accounted for in the design and analysis? | Can’t tell |  |
|  | 3.5. During the study period, is the intervention administered (or exposure occurred) as intended? | Yes |  |
| McCalla et al. 2018 | 3.1. Are the participants representative of the target population? | Yes | 100% |
|  | 3.2. Are measurements appropriate regarding both the outcome and intervention (or exposure)? | Yes |  |
|  | 3.3. Are there complete outcome data? | Yes |  |
|  | 3.4. Are the confounders accounted for in the design and analysis? | Yes |  |
|  | 3.5. During the study period, is the intervention administered (or exposure occurred) as intended? | Yes |  |
| Benudis et al. 2019 | 3.1. Are the participants representative of the target population? | Yes | 80% |
|  | 3.2. Are measurements appropriate regarding both the outcome and intervention (or exposure)? | Yes |  |
|  | 3.3. Are there complete outcome data? | Yes |  |
|  | 3.4. Are the confounders accounted for in the design and analysis? | Can’t tell |  |
|  | 3.5. During the study period, is the intervention administered (or exposure occurred) as intended? | Yes |  |

**Table A3. MMAT for quantitative descriptive study.**

| **Reference** | **MMAT item** | **Yes/No** | **MMAT score** |
| --- | --- | --- | --- |
| Azim et al. 2016 | 4.1. Is the sampling strategy relevant to address the research question? | Yes | 100% |
|  | 4.2. Is the sample representative of the target population? | Yes |  |
|  | 4.3. Are the measurements appropriate? | Yes |  |
|  | 4.4. Is the risk of nonresponse bias low? | Yes |  |
|  | 4.5. Is the statistical analysis appropriate to answer the research question? | Yes |  |
| Helder et al. 2012 | 4.1. Is the sampling strategy relevant to address the research question? | Yes | 100% |
|  | 4.2. Is the sample representative of the target population? | Yes |  |
|  | 4.3. Are the measurements appropriate? | Yes |  |
|  | 4.4. Is the risk of nonresponse bias low? | Yes |  |
|  | 4.5. Is the statistical analysis appropriate to answer the research question? | Yes |  |
| Levchenko et al. 2013 | 4.1. Is the sampling strategy relevant to address the research question? | Yes | 100% |
|  | 4.2. Is the sample representative of the target population? | Yes |  |
|  | 4.3. Are the measurements appropriate? | Yes |  |
|  | 4.4. Is the risk of nonresponse bias low? | Yes |  |
|  | 4.5. Is the statistical analysis appropriate to answer the research question? | Yes |  |
| Cheng et al. 2011 | 4.1. Is the sampling strategy relevant to address the research question? | Yes | 80% |
|  | 4.2. Is the sample representative of the target population? | Yes |  |
|  | 4.3. Are the measurements appropriate? | Yes |  |
|  | 4.4. Is the risk of nonresponse bias low? | Can’t tell |  |
|  | 4.5. Is the statistical analysis appropriate to answer the research question? | Yes |  |
| AI Salman et al. 2015 | 4.1. Is the sampling strategy relevant to address the research question? | Yes | 80% |
|  | 4.2. Is the sample representative of the target population? | Yes |  |
|  | 4.3. Are the measurements appropriate? | Yes |  |
|  | 4.4. Is the risk of nonresponse bias low? | No |  |
|  | 4.5. Is the statistical analysis appropriate to answer the research question? | Yes |  |
| Dyson and Madeo 2017 | 4.1. Is the sampling strategy relevant to address the research question? | Yes | 80% |
|  | 4.2. Is the sample representative of the target population? | Yes |  |
|  | 4.3. Are the measurements appropriate? | Yes |  |
|  | 4.4. Is the risk of nonresponse bias low? | No |  |
|  | 4.5. Is the statistical analysis appropriate to answer the research question? | Yes |  |
| Gould et al. 2020 | 4.1. Is the sampling strategy relevant to address the research question? | Yes | 100% |
|  | 4.2. Is the sample representative of the target population? | Yes |  |
|  | 4.3. Are the measurements appropriate? | Yes |  |
|  | 4.4. Is the risk of nonresponse bias low? | Yes |  |
|  | 4.5. Is the statistical analysis appropriate to answer the research question? | Yes |  |
| Iversen et al. 2020 | 4.1. Is the sampling strategy relevant to address the research question? | Yes | 80% |
|  | 4.2. Is the sample representative of the target population? | Yes |  |
|  | 4.3. Are the measurements appropriate? | Yes |  |
|  | 4.4. Is the risk of nonresponse bias low? | Can’t tell |  |
|  | 4.5. Is the statistical analysis appropriate to answer the research question? | Yes |  |
| Monsalve et al. 2014 | 4.1. Is the sampling strategy relevant to address the research question? | Yes | 80% |
|  | 4.2. Is the sample representative of the target population? | Yes |  |
|  | 4.3. Are the measurements appropriate? | Yes |  |
|  | 4.4. Is the risk of nonresponse bias low? | Can’t tell |  |
|  | 4.5. Is the statistical analysis appropriate to answer the research question? | Yes |  |
| Hagel et al. 2015 | 4.1. Is the sampling strategy relevant to address the research question? | Yes | 100% |
|  | 4.2. Is the sample representative of the target population? | Yes |  |
|  | 4.3. Are the measurements appropriate? | Yes |  |
|  | 4.4. Is the risk of nonresponse bias low? | Yes |  |
|  | 4.5. Is the statistical analysis appropriate to answer the research question? | Yes |  |
| McLaws et al. 2018 | 4.1. Is the sampling strategy relevant to address the research question? | Yes | 100% |
|  | 4.2. Is the sample representative of the target population? | Yes |  |
|  | 4.3. Are the measurements appropriate? | Yes |  |
|  | 4.4. Is the risk of nonresponse bias low? | Yes |  |
|  | 4.5. Is the statistical analysis appropriate to answer the research question? | Yes |  |
| Filho et al. 2014 | 4.1. Is the sampling strategy relevant to address the research question? | Yes | 100% |
|  | 4.2. Is the sample representative of the target population? | Yes |  |
|  | 4.3. Are the measurements appropriate? | Yes |  |
|  | 4.4. Is the risk of nonresponse bias low? | Yes |  |
|  | 4.5. Is the statistical analysis appropriate to answer the research question? | Yes |  |
| McCalla et al. 2017 | 4.1. Is the sampling strategy relevant to address the research question? | Yes | 100% |
|  | 4.2. Is the sample representative of the target population? | Yes |  |
|  | 4.3. Are the measurements appropriate? | Yes |  |
|  | 4.4. Is the risk of nonresponse bias low? | Yes |  |
|  | 4.5. Is the statistical analysis appropriate to answer the research question? | Yes |  |
| Boyce et al. 2019 | 4.1. Is the sampling strategy relevant to address the research question? | Yes | 100% |
|  | 4.2. Is the sample representative of the target population? | Yes |  |
|  | 4.3. Are the measurements appropriate? | Yes |  |
|  | 4.4. Is the risk of nonresponse bias low? | Yes |  |
|  | 4.5. Is the statistical analysis appropriate to answer the research question? | Yes |  |
| Srigley et al. 2014 | 4.1. Is the sampling strategy relevant to address the research question? | Yes | 100% |
|  | 4.2. Is the sample representative of the target population? | Yes |  |
|  | 4.3. Are the measurements appropriate? | Yes |  |
|  | 4.4. Is the risk of nonresponse bias low? | Yes |  |
|  | 4.5. Is the statistical analysis appropriate to answer the research question? | Yes |  |
|  |  |  |  |
|  |  |  |  |
|  |  |  |  |
|  |  |  |  |
|  |  |  |  |
| Conway et al. 2014 | 4.1. Is the sampling strategy relevant to address the research question? | Yes | 100% |
|  | 4.2. Is the sample representative of the target population? | Yes |  |
|  | 4.3. Are the measurements appropriate? | Yes |  |
|  | 4.4. Is the risk of nonresponse bias low? | Yes |  |
|  | 4.5. Is the statistical analysis appropriate to answer the research question? | Yes |  |
| Levchenko et al. 2011 | 4.1. Is the sampling strategy relevant to address the research question? | Yes | 100% |
|  | 4.2. Is the sample representative of the target population? | Yes |  |
|  | 4.3. Are the measurements appropriate? | Yes |  |
|  | 4.4. Is the risk of nonresponse bias low? | Yes |  |
|  | 4.5. Is the statistical analysis appropriate to answer the research question? | Yes |  |
| Edmisten et al. 2017 | 4.1. Is the sampling strategy relevant to address the research question? | Yes | 100% |
|  | 4.2. Is the sample representative of the target population? | Yes |  |
|  | 4.3. Are the measurements appropriate? | Yes |  |
|  | 4.4. Is the risk of nonresponse bias low? | Yes |  |
|  | 4.5. Is the statistical analysis appropriate to answer the research question? | Yes |  |
